# Diagnostic value of HMGB-1 and acetylcholinesterase in assessing the prognosis of patients with acute pancreatitis

**DOI:** 10.5937/jomb0-53786

**Published:** 2025-06-13

**Authors:** Guofei Peng, Wanfang Chen, Yan Li, Danping Zhang

**Affiliations:** 1 Huanggang Central Hospital, Department of Clinical Laboratory, Huanggang, Hubei; 2 Huanggang Central Hospital, Department of Blood Transfusion, Huanggang, Hubei

**Keywords:** acetylcholinesterase, acute pancreatitis, high mobility group box 1 protein, nutritional state, prognostic assessment, acetilholinesteraza, akutni pankreatitis, protein 1 iz grupe proteina visoke mobilnosti (HMGB-1), nutritivno stanje, procena prognoze

## Abstract

**Background:**

Acute pancreatitis (AP) is a disorder of tissue digestion caused by abnormal activation of pancreatic enzymes, which may lead to multi-organ failure and, ultimately, death as the disease progresses. How to quickly and accurately evaluate the progression of AP has always been a hotspot and difficulty in clinical research.

**Methods:**

Sixty-four AP patients were admitted to our hospital between August 2022 and June 2023, and 60 healthy people during the same period were selected for retrospective analysis, with AP patients as the observation group and healthy people as the control group. HMGB-1 and AChE levels were detected in both groups, and the diagnostic value of HMGB-1 and AChE for AP was analysed using the receiver operating characteristic (ROC) curve. Subsequently, the differences in the expression of HMGB-1 and AChE in AP patients with different severities were further observed. In addition, we detected albumin (ALB), transferrin (TRF), and total protein (TP) in the observation group and analysed their correlation with HMGB-1 and AChE. Finally, a 6-month prognostic follow-up was performed to analyse the predictive value of HMGB-1 and AChE for poor prognosis in AP using ROC curves.

**Results:**

Compared with the control group, HMGB-1 was higher in the observation group, which was positively correlated with the severity of AP (P<0.05), while AChE was lower, which was negatively correlated with the severity of AP (P<0.05). HMGB-1+AChE had a sensitivity of 48.44% and a specificity of 88.33% for diagnosing AP (P<0.05, cut-off>0.639). In addition, HMGB-1 and nutrient proteins were positively correlated, and AChE and nutrient proteins were negatively correlated in the observation group (P<0.05). The prognostic follow-up showed that the diagnostic sensitivity of HMGB-1+AChE for poor prognosis of AP was 95.65%, and the specificity was 65.85% (P<0.05).

**Conclusions:**

HMGB-1 was elevated in AP, and AChE was decreased in AP, both of which have excellent diagnostic effects on the occurrence and poor prognosis of AP.

## Abstract (sr)

**Uvod:**

Akutni pankreatitis (AP) je poremećaj varenja tkiva uzrokovan abnormalnom aktivacijom enzima pankreasa, koji može dovesti do otkazivanja više organa i, u krajnjem ishodu, smrti kako bolest napreduje. Brza i precizna procena napretka AP-a uvek je bila važna i izazovna tema u kliničkim istraživanjima.

**Metode:**

šezdeset četiri pacijenta sa akutnim pankreatitisom (AP) su primljena u našu bolnicu izme|u avgusta 2022. i juna 2023. godine, a za retrospektivnu analizu je odabrano 60 zdravih osoba tokom istog perioda, pri čemu su AP pacijenti činili posmatranu grupu, a zdrave osobe kontrolnu. U obe grupe su mereni nivoi HMGB-1 i AChE, a dijagnostička vrednost HMGB-1 i AChE za AP je analizirana korišćenjem ROC krive. Zatim su dalje posmatrane razlike u ekspresiji HMGB-1 i AChE kod pacijenata sa AP koji su bili različite težine. Pored toga, u posmatranoj grupi mereni su albumin (ALB), transferin (TRF) i ukupni protein (TP) i analizirana njihova korelacija sa HMGB-1 i AChE. Na kraju je izvršeno praćenje prognoze tokom 6 meseci radi analize prediktivne vrednosti HMGB-1 i AChE za lošu prognozu kod AP korišćenjem ROC krivih.

**Rezultati:**

U poređenju sa kontrolnom grupom, HMGB-1 je bio povišen u posmatranoj grupi i pozitivno je korelirao sa težinom AP (P<0,05), dok je AChE bio niži i negativno korelirao sa težinom AP (P<0,05). Kombinovani pokazatelj HMGB-1+AChE je imao osetljivost od 48,44% i specifičnost od 88,33% za dijagnozu AP (P<0,05, granična vrednost >0,639). Pored toga, HMGB-1 i nutritivni proteini bili su u pozitivnoj korelaciji, dok su AChE i nutritivni proteini bili u negativnoj korelaciji u posmatranoj grupi (P<0,05). Praćenje prognoze je pokazalo da je dijagnostička osetljivost kombinovanog pokazatelja HMGB-1+AChE za lošu prognozu AP iznosila 95,65%, a specifičnost 65,85% (P<0,05).

**Zaključak:**

HMGB-1 je bio povišen kod AP, a AChE je bio smanjen kod AP, a oba pokazatelja imaju odličan dijagnostički efekat na pojavu i lošu prognozu AP.

## Introduction

Acute pancreatitis (AP), a sudden disease of pancreatic self-digestion caused by abnormal activation of pancreatic enzymes and a very common digestive system disease, has an annual incidence of about 50–30/100,000 and a rising morbidity year by year [1]. The major symptoms of AP are sudden and persistent epigastric pain, which may be accompanied by nausea, vomiting, abdominal distension, fever, etc. In more severe cases, AP may cause multiple organ dysfunction and lead to death [2]. Due to the acute onset, short course of disease, and rapid progression, effectively evaluating the progression of AP is the key issue in ensuring patients’ health. However, there is currently no stable early prediction and prognosis assessment plan for AP in clinical practice, and the diagnosis of AP usually requires a comprehensive evaluation of clinical symptoms, blood tests, imaging examinations, and other methods [3]. These limitations have resulted in a high incidence of AP and a consistent mortality of 5–15% [4]. Finding an accurate and fast indicator for evaluating the condition of AP is, therefore, the hotspot and difficulty of modern clinical research.

High mobility group box 1 protein (HMGB-1) is a new type of pro-inflammatory cytokine discovered in recent years. It has attracted extensive attention in critical care medicine research due to its important inflammatory regulatory role [5] [6]. For AP, HMGB-1 has also been shown to be of great significance [7]. However, as a broad pro-inflammatory substance, HMGB-1 is not only related to AP but may also be influenced by other inflammatory reactions in the human body. Therefore, evaluating AP solely through HMGB-1 still lacks certain specificity. Acetylcholine, released by cholinergic neurons, is the primary parasympathetic neurotransmitter, which can enhance neuronal excitability in response to external stress stimuli, leading to neuronal apoptosis and neurological injury [8]. The cholinergic nerve system is involved in the physiological processes of many diseases. For example, many previous studies have associated cholinesterase with disease severity and prognosis [9] [10]. However, most current research focuses on butyrylcholinesterase, with extremely rare reports on acetylcholinesterase (AChE). Research has shown that in high glucose environments, HMGB-1 can exert a synergistic effect through AChE, stimulating the sensitivity of neuronal cells and causing pain [11]. Meanwhile, an animal study by Meng Q et al. even mentioned that regulating HMGB-1 and AChE in mice has a synergistic effect, and interfering with the expression of both can regulate blood glucose in mice [12]. These results suggest a potential link between HMGB-1 and AChE, and the potential of their combination to improve the evaluation of AP greatly.

Studies on HMGB-1 and AChE in AP are still rare, and clinical references are still lacking to determine the clinical role of HMGB-1 and AChE in AP. In this study, we will initially analyse the clinical significance of HMGB-1 and AChE in AP, and these results may provide new references and guidelines for the future diagnosis and treatment of AP.

## Materials and methods

### Research subjects

The sample size required for this study was calculated using PASS software with α=0.5, which showed that a minimum of 45 study subjects were required. Sixty-four AP patients were admitted to our hospital between August 2022 and June 2023, and 60 healthy people during the same period were selected for retrospective analysis, with AP patients as the observation group and healthy people as the control group. Ethical approval has been obtained from the Ethics Committee of our hospital, and all subjects have provided informed consent.

### Eligibility and exclusion criteria

Observation group: The included patients, all aged between 18 and 60, met the AP diagnostic guidelines [13] and were confirmed by various tests after admission, with first onset, complete medical records, and no surgical treatment. Patients with severe organ dysfunction were excluded; patients with immunodeficiency were excluded; patients unable to communicate normally were excluded; patients in coma were excluded; patients with comorbid pancreatic cancer were excluded; and patients with comorbid infectious diseases were excluded [14]. Control group: Healthy controls aged 18–60 who underwent physical examinations in our hospital, with complete medical records, no previous major medical history, and normal physical examination results, were included. The exclusion criteria were the same as in the observation group.

### Methods

4 mL of fasting venous blood was drawn from AP patients within 24 hours after admission and from healthy controls on the day of physical examination, and the serum was isolated via centrifugation and stored in a -80°C refrigerator. Using kits from Wuhan Fine Biotechnology Co., Ltd., an enzyme-linked immunosorbent assay (ELISA) was carried out to measure HMGB-1 and AChE levels. In addition, an automatic biochemical analyser was utilised to quantify the levels of nutritional proteins albumin (ALB), transferrin (TRF), and total protein (TP) in the observation group.

### Condition assessment

Patients in the observation group were examined by computerised tomography (CT) on the 3rd day after onset, and the image results were crossevaluated by two radiologists. The progression of AP was judged based on the modified CT severity index (MCTSI) [15]: 0–2, 4–6, and 8–10 points were rated as mild (MAP), moderation to severe (MSAP), and severe AP (SAP), respectively.

### Follow-up for prognosis

Patients in the observation group underwent a 6-month follow-up after discharge, which was conducted as regular follow-up, with an interval of no more than 1 month. The occurrence of multiple organ dysfunction syndrome, pancreatic necrosis, or death during the follow-up period was regarded as a poor prognosis; otherwise, the prognosis was considered good.

### Outcome measures

The diagnostic value of HMGB-1 and AChE in AP and their correlations with pathological progression were discussed; the correlation of HMGB-1 and AChE with nutritional proteins in the observation group was analysed, and the prognosis of patients in the observation group was statistically analysed, and the prognosis evaluation effect of HMGB-1 and AChE on AP was analysed.

### Statistical analysis

SPSS v24.0 software was used for statistical analysis. Chi-square tests were performed to identify differences between two groups of counting data represented by [n(%)]. Independent sample t-tests were used for inter-group comparisons of measurement data represented by ( ±s), comparisons between multiple data sets were made using repeated measures ANOVA and Least-Significant Difference withingroup tests. The diagnostic value was analysed by receiver operating characteristic (ROC) curves, and the diagnostic effect was evaluated by the area under the curve (AUC); the closer the AUC is to 1, the more accurate the diagnosis will be. For the combined detection, the joint detection formula Log(P) was obtained by binary Logistic regression analysis, and then ROC analysis was performed. Correlation analysis was conducted using Pearson and Spearman correlation coefficients. A minimum significance threshold of P<0.05 was used.

## Results

### Comparison of clinical data

By comparison, we found no significant difference between the observation and control groups in terms of age, sex, family history, etc. (P>0.05), confirming their comparability (Table 1).

**Table 1:** Comparison of clinical data.

Groups	Male	Female	Age	Etiology			
				Biliary	Alcoholic	Hyperlipidaemic	Other
Control (n=60)	39 (65.00)	21 (35.00)	45.08±3.55	-	-	-	-
Observation (n=64)	40 (62.50)	24 (37.50)	45.56±4.30	33 (51.56)	12 (18.75)	16 (25.00)	3 (4.69)
χ^2^ (t)	0.084	0.674					
P	0.772	0.502					

### Diagnostic efficacy of HMGB-1 and AChE for AP

**Figure 1:**
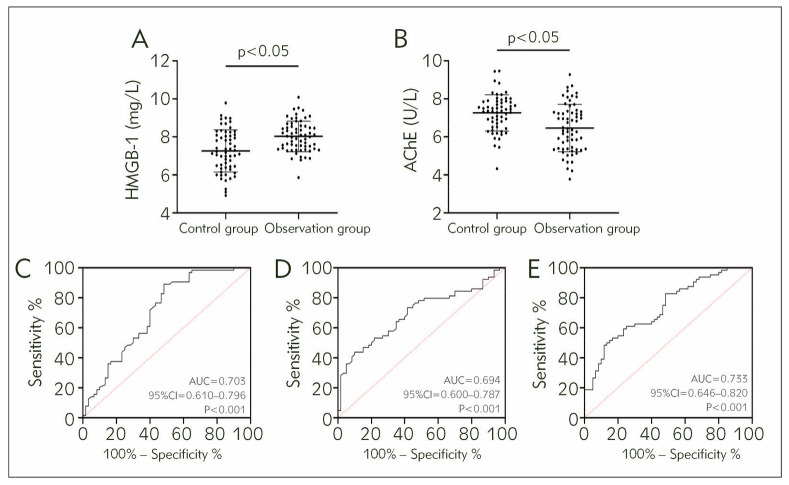
Diagnostic efficacy of HMGB-1 and AChE for AP<br>A) Comparison of HMGB-1, B) Comparison of AChE, C) ROC curves for HMGB-1 diagnostic AP, D) ROC curves for AChE diagnostic AP. E) ROC curve of HMGB-1 combined with AChE for diagnosis of AP. High mobility group box 1 protein, HMGB-1; Acetylcholinesterase, AChE; Receiver operating characteristic, ROC; Area under the curve, AUC.

The HMGB-1 in the observation group was higher than in the control group, while the AChE was lower (P<0.05). ROC curve analysis showed that when HMGB-1 was less than 7.25 mg/L, the sensitivity and specificity for diagnosing AP were 89.06% and 51.67%, respectively (P<0.05); when AChE was less than 6.18 U/L, the sensitivity for diagnosing AP was 43.75%, and the specificity was 90.00% (P<0.05). Using the joint detection Log(P)=-1.736+(0.645× HMGB-1)+(-0.454×AChE), it was found that HMGB-1+AChE had a sensitivity of 48.44% and a specificity of 88.33% for the diagnosis of AP (P<0.05, cut-off >0.639, Figure 1).

A) Comparison of HMGB-1, B) Comparison of AChE, C) ROC curves for HMGB-1 diagnostic AP, D) ROC curves for AChE diagnostic AP. E) ROC curve of HMGB-1 combined with AChE for diagnosis of AP. High mobility group box 1 protein, HMGB-1; Acetyl cholinesterase, AChE; Receiver operating characteristic, ROC; Area under the curve, AUC.

### Relationship between HMGB-1, AChE, and AP progression

**Figure 2:**
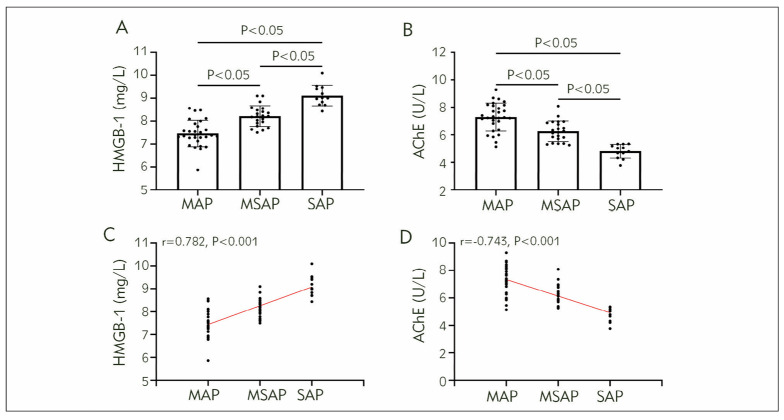
Relationship between HMGB-1, AChE, and AP progression.<br>A) Comparison of HMGB-1, B) Comparison of AChE, C) Correlation between HMGB-1 and AP progression, D) Correlation between AChE and AP progression. High mobility group box 1 protein, HMGB-1; Acetylcholinesterase, AChE; Mild acute pancreatitis, MAP; Moderation to severe acute pancreatitis, MSAP; Severe acute pancreatitis, SAP.

In the observation group, SAP patients showed higher HMGB-1 levels than MAP and MSAP patients, and MSAP patients had higher HMGB-1 levels than MAP patients (P<0.05). The AChE of SAP patients was the lowest, and that of MAP patients was higher compared with MSAP patients (P<0.05). Spearman correlation coefficient analysis showed that HMGB-1 was positively correlated with AP progression while AChE was negatively correlated with disease progression in the observation group (P<0.05, Figure 2).

A) Comparison of HMGB-1, B) Comparison of AChE, C) Correlation between HMGB-1 and AP progression, D) Correlation between AChE and AP progression. High mobility group box 1 protein, HMGB-1; Acetylcholinesterase, AChE; Mild acute pan crea titis, MAP; Moderation to severe acute pancreatitis, MSAP; Severe acute pancreatitis, SAP.

### Association between HMGB-1, AChE, and nutritional status in AP patients

**Figure 3:**
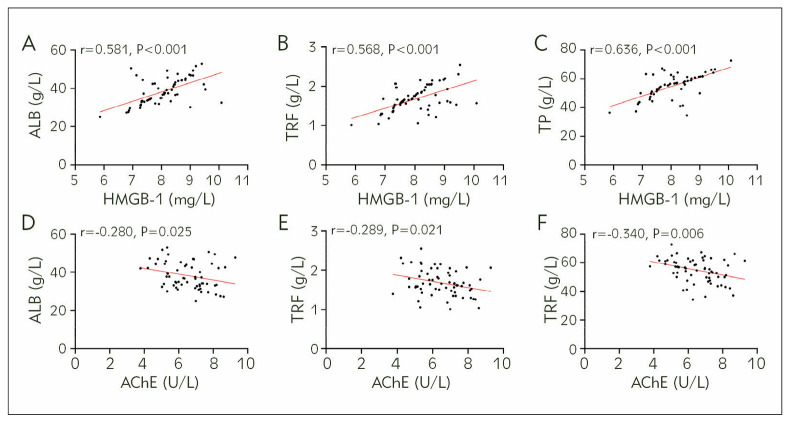
Association between HMGB-1, AChE, and nutritional status in AP patients.<br>A) Correlation analysis of HMGB-1 and ALB, B) Correlation analysis of HMGB-1 and TRF, C) Correlation analysis of HMGB-1 and TP, D) Correlation analysis of AChE and ALB, E) Correlation analysis of AChE and TRF, F) Correlation analysis of AChE and TP. High mobility group box 1 protein, HMGB-1; Acetylcholinesterase, AChE; Albumin, ALB; Transferrin, TRF; Total protein, TP.

After testing, the ALB, TRF, and TP of the observation group were (38.28±6.74) g/L, (1.68±0.33) g/L, and (54.80±8.31) g/L, respectively. According to Pearson correlation coefficient analysis, ALB, TRF, and TP were inversely associated with HMGB-1 in the observation group and positively correlated with AChE (P<0.05, Figure 3).

A) Correlation analysis of HMGB-1 and ALB, B) Cor relation analysis of HMGB-1 and TRF, C) Cor relation analysis of HMGB-1 and TP, D) Correlation analysis of AChE and ALB, E) Correlation analysis of AChE and TRF, F) Correlation analysis of AChE and TP. High mobility group box 1 protein, HMGB-1; Acetylcholinesterase, AChE; Albumin, ALB; Transfer rin, TRF; Total protein, TP.

### Effect of HMGB-1 and AChE on prognosis evaluation of AP patients

**Figure 4:**
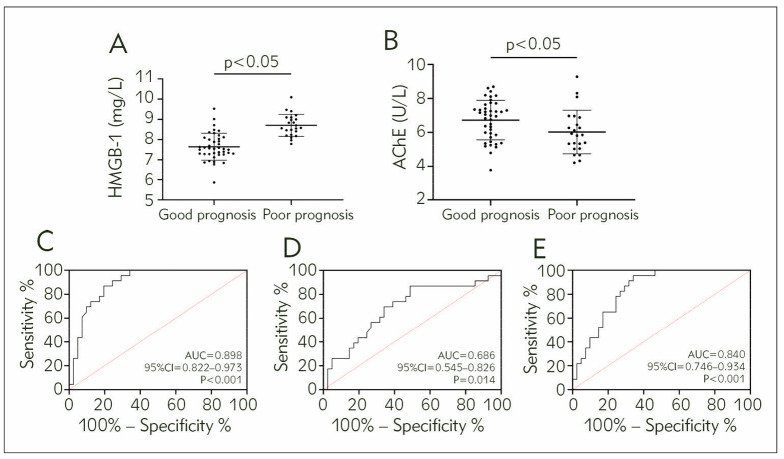
Effect of HMGB-1 and AChE on prognosis evaluation of AP patients<br>A) Comparison of HMGB-1, B) Comparison of AChE, C) ROC curve for HMGB-1 diagnosis of poor prognosis in AP, D) ROC curve for AChE diagnosis of poor prognosis in AP, E) ROC curve for HMGB-1 and AChE diagnosis of poor prognosis in AP.<br>High mobility group box 1 protein, HMGB-1; Acetylcholinesterase, AChE; Receiver operating characteristic, ROC; Area under the curve, AUC

Based on the follow-up results, 41 patients were included in the good prognosis group and 23 patients in the poor prognosis group. Through comparison, it was found that the good prognosis group had lower HMGB-1 and higher AChE levels than the poor prognosis group (P<0.05). ROC curve analysis revealed that HMGB-1 had a sensitivity of 86.96% and a specificity of 80.49% in predicting poor prognosis (P<0.05, cut-off>8.15 mg/L). The sensitivity of AChE in predicting adverse prognosis was 86.96%, and the specificity was 51.22% (P<0.05, cut-off<6.98 U/L). The combined detection of Log (P)=-8.026+(1.028×HMGB-1)+(-0.382×AChE) showed a sensitivity of 95.65% and a specificity of 65.85% for predicting poor prognosis in AP patients (P<0.05, cut-off >0.074, Figure 4).

A) Comparison of HMGB-1, B) Comparison of AChE, C) ROC curve for HMGB-1 diagnosis of poor prognosis in AP, D) ROC curve for AChE diagnosis of poor prognosis in AP, E) ROC curve for HMGB-1 and AChE diagnosis of poor prognosis in AP

High mobility group box 1 protein, HMGB-1; Acetylcholinesterase, AChE; Receiver operating characteristic, ROC; Area under the curve, AUC.

## Discussion

In this study, we found that both HMGB-1 and AChE were significantly aberrantly expressed in AP. Both demonstrated excellent effects on the assessment of the disease of AP, and these findings may provide new references for future clinics in understanding the progression of AP.

First, the levels of HMGB-1 and AChE were compared between the observation and control groups. HMGB-1 was found to be increased in the observation group while AChE decreased, suggesting that the two may be involved in the occurrence and development of AP. Similarly, a study by Yang J et al. [16] proposed central mediators of HMGB-1 pancreatitis that lead to worsening inflammation and inflammatory deterioration in pancreatitis, which is consistent with our results. However, for AChE, although there is no direct evidence of AChE expression in AP, we found that in a targeted drug study on hepatocellular carcinoma by Wang K et al. [17], they found that elevated AChE as a therapeutic target could effectively improve the progression of hepatocellular carcinoma. These results also corroborate the organ functioning Regulatory effect of AChE on organ function. Clinical research shows that HMGB-1 can be secreted passively by injured and necrotic cells or actively by activated immune cells under the stimulation of cytokines, while it can also be released in large quantities in various pathological processes such as sepsis, tumours, infections, and shock [18]. Previous research has also preliminarily validated the relationship between HMGB-1 and AP, supporting the two as key factors in the future diagnosis and treatment of AP [16]. However, the exact relationship between HMGB-1 and AP has not been verified. AChE, a key enzyme in biological nerve conduction with high activity in serum, can degrade acetylcholine, ensure normal transmission of nerve signals, and participate in the physiological processes of various diseases [19]. In AP, a series of peroxidation damage, stress injury, and even necrosis caused by the massive release of inflammatory mediators in organs and tissues may inhibit the secretion of AChE, which is speculated to be the main reason for the decrease of AChE in the observation group. Through ROC curve analysis, both HMGB-1 and AChE exhibited excellent diagnostic effects for AP, with the AUC, sensitivity, and specificity of their combined diagnosis being 0.733, 48.44%, and 88.33%, respectively. Contrast-enhanced CT scanning, the widely recognised and major diagnostic approach for AP in the current clinical practice, can correctly judge the patient’s condition and inflammatory response levels. However, it is limited by the inability to achieve a wide range of clinical screening and the possible influence of the examination results by certain subjective factors [20].

On the other hand, the detection of HMGB-1 and AChE is rapid, convenient, and objective, with blood as the detection sample, which can greatly improve the ability of large-scale screening and early evaluation of AP, showing high clinical application value. In a study by Wu W et al., they found a close relationship between HMGB-1 and neutrophil dysfunction in patients with HBV-induced liver failure and that HMGB-1 has the potential to be an indicator for assessing the condition of liver failure [21]. As for AChE, the study by Beltman L et al. attempting to diagnose congenital megacolon by detecting AChE also achieved remarkable results [22]. They concluded that there is a close link between AChE and inflammatory response; therefore, it can effectively respond to inflammatory damage of organs in the human body [22]. These studies lay the foundation for future clinical applications of HMGB-1 and AChE. Meanwhile, we found a positive correlation between HMGB-1 and AP progression in the observation group and a negative association between AChE and disease progression, demonstrating the close relationship between the two and AP progression and their potential as clinical indicators of AP.

As mentioned earlier, the main pathology of AP is the activation of self-digestion by pancreatic enzymes, which consequently results in changes in vital signs such as metabolic disorders of carbohydrates, proteins, and fats (the three primary macronutrients) in tissues, water-electrolyte imbalance, and acid-base disturbance [23]. Therefore, the nutritional status of AP patients is the basis for ensuring therapeutic effectiveness and the key to avoiding treatment-associated complications [24]. In previous studies, metabolic disorders and nutrient protein loss in AP patients have been unanimously recognised clinically [25]. In this study, patients in the observation group showed markedly reduced ALB, TRF, and TP levels compared to the normal reference values, which aligns with previous studies results. Through correlation analysis, it can be seen that HMGB-1 in the observation group was negatively correlated with ALB, TRF, and TP. At the same time, AChE was positively related to these indicators, suggesting the close correlation of HMGB-1 and AChE with nutritional status in AP patients and further supporting their involvement in AP progression. The reason for this is believed to be consistent with the above inference; that is, the intensified inflammatory response caused by HMGB-1 inhibits the secretion of AChE, which enhances the self-digestion ability of pancreatic enzymes and worsens metabolic disorders in patients, resulting in a significant loss of nutritional proteins. Hass U et al. showed a negative correlation between HMGB-1 levels and muscle strength in older adults [26], which is consistent with our view. In a food chemistry study by Romero-Márquez JM et al. [27], they also mentioned that food is more favourable for human nutrition and health if it contains anti-AChE substances. However, more research is needed to confirm the relationship between HMGB-1, AChE, and the nutritional status of AP patients.

Finally, in the prognostic follow-up, higher HMGB-1 and lower AChE were found in patients with poor prognosis, which showed excellent evaluation effects on poor outcomes in AP. It suggests that the prognosis of patients can be evaluated as soon as possible by detecting HMGB-1 and AChE in the future, thus allowing for timely interventions to ensure the prognosis and health of patients.

## Conclusion

HMGB-1 is increased in AP, and AChE is decreased, both of which show excellent diagnostic effects on the occurrence of AP and poor prognosis and are closely related to the nutritional status of patients. In the future, we can monitor the levels of HMGB-1 and AChE to evaluate the development of AP and implement intervention measures in time to ensure the prognosis and health of patients. However, since the changes in HMGB-1 and AChE before and after treatment in AP patients were not measured in this study, we are not sure whether the levels of HMGB-1 and AChE will be affected by treatment. We believe that HMGB-1 and AChE after treatment have a more intuitive evaluation effect on the prognosis of AP, which needs to be confirmed by further studies. In addition, more in-depth experiments are required to confirm further the exact mechanism of action of HMGB-1 and AChE in AP and provide a more reliable reference for clinical practice.

## Dodatak

### Ethical Approval

The study involving human subjects complied with the Declaration of Helsinki. It was approved by the ethical committee of the Huanggang Central Hospital (Approval number: 2022-L132), and all participants provided written informed consent.

### Availability of data and materials

The data used to support the findings of this study are available from the corresponding author upon request.

### Contribution of authors

Yan Li conceived and designed the project, and Guofei Peng wrote the manuscript. Danping Zhang generated and analysed the data. Wanfang Chen modified the manuscript, and Guofei Peng and Wanfang Chen contributed equally to this work as cofirst authors. All authors gave final approval of the version to be published.

### Conflict of interest statement

All the authors declare that they have no conflict of interest in this work.
